# Pulmonary Function after Proton Therapy for Hodgkin Lymphoma

**DOI:** 10.14338/IJPT-18-00040.1

**Published:** 2019-03-21

**Authors:** Lillie O'steen, Jason Bellardini, James Cury, Lisa Jones, Vandana K. Seeram, Nancy P. Mendenhall, Bradford S. Hoppe

**Affiliations:** 1 Department of Radiation Oncology, University of Florida College of Medicine, Gainesville and Jacksonville, FL, USA; 2 Department of Medicine, University of Florida College of Medicine, Jacksonville, FL, USA

**Keywords:** radiation therapy, proton therapy, chemotherapy, Hodgkin lymphoma, pulmonary function tests

## Abstract

**Purpose:**

Acute and late toxicity from chemotherapy, targeted therapy, and radiation therapy can cause significant morbidity among survivors of Hodgkin lymphoma (HL), including pulmonary dysfunction. Improved dosimetry may influence pulmonary function tests (PFTs), an objective and clinically significant measure of pulmonary toxicity. The present study investigates the impact of proton therapy on PFTs among HL survivors.

**Patients and Methods:**

We monitored 15 patients with mediastinal HL who were enrolled in an institutional HL trial. All patients were treated with combination chemotherapy plus involved-node proton therapy. All patients were to undergo PFTs before starting treatment and at approximately 6 and 12 months after completing proton therapy.

**Results:**

Twelve patients were included in the analysis and 3 excluded. The mean forced vital capacity (FVC) was 96.2% ± 16.5% (mean ± SD) predicted at baseline and 98.2% ± 19.4% predicted at 12 months. The mean forced expiratory volume in 1 second (FEV_1_) was 96.7% ± 17.2% predicted at baseline and 97% ± 15.1% predicted at 12 months. The mean FEV_1_/FVC ratio was 99.5 ± 8.29 at baseline and 97.8 ± 8.02 at 12 months. The mean diffusing capacity of the lung for carbon monoxide was 81.4% ± 18.4% predicted at baseline and 95.7% ± 23.5% predicted at 12 months.

**Conclusion:**

No unexpected changes were observed to the lungs as illustrated through follow-up PFTs. Long-term follow-up and validation in a larger cohort are needed.

## Introduction

Combined modality therapy with chemotherapy and radiation provide excellent disease control among patients with Hodgkin lymphoma (HL). However, the acute and late toxicities from chemotherapy, targeted therapy, and radiation therapy (RT) can cause significant morbidity among survivors, including pulmonary dysfunction [1–4].

Rates of abnormal pulmonary function test (PFT) findings have been reported to be as high as 32% in survivors of HL treated with photon-based involved-field RT [1] and 56% when more extensive fields are included [2]. Abnormalities in PFTs have been associated with decreased overall survival in large studies of the general population [5].

Utilization of more conformal RT techniques, such as proton therapy, may reduce the risk of these late side effects. Dosimetric studies of 3-dimensional (3D) proton therapy have demonstrated a reduction in mean lung dose as compared to x-ray–based 3D conformal RT and intensity-modulated RT [6]. Volumes of lung receiving 5 to 30 Gy can be reduced up to 46% when compared to 3D conformal RT and intensity-modulated RT [7]. A review of 59 patients who received proton radiation therapy for mediastinal lymphoma demonstrated a low incidence of acute and late pulmonary toxicity evaluated clinically during treatment and follow-up visits [12]. However, no studies to date have evaluated how the improved dosimetric values of proton therapy influence PFTs, an objective and clinically significant measure of pulmonary toxicity. The present study investigates the impact of proton therapy on PFTs among HL survivors.

## Materials and Methods

We monitored 15 patients with mediastinal HL who were enrolled in the prospective trial Proton Therapy for Hodgkin Lymphoma (HL01) and treated at the University of Florida Health Proton Therapy Institute (Jacksonville, Florida) beginning in September 2009. All patients were treated with combination chemotherapy plus involved-node proton therapy. Details of treatment planning and doses to the organs at risk for this cohort have previously been reported by our institution. All patients were to undergo PFTs before starting treatment and at approximately 6 and 12 months after completing proton therapy.

## Results

We identified 12 patients for analysis. Three patients were unable to complete initial or follow-up PFTs because of young age (7 years old) (n = 1), recurrence (n = 1), and diagnosis of another cancer (n = 1). All patients received combination chemotherapy plus proton therapy. Patient and treatment characteristics are detailed in **Table 1**.

**Table 1.** Patient and treatment characteristics (N = 12).

**Characteristic**	**Value (%)**
Female	9 (75) pts
Bulky mediastinal disease	10 (83) pts
History of smoking	0 (0) pts
Median age	27.9 y
Chemotherapy	
ABVD × 4 cycles	5 (42) pts
ABVD × 6 cycles	4 (33) pts
ABVE-PC × 4 cycles	3 (25)
Radiation therapy	
Protons only	11 (92)
Protons and photons	1 (8)
Median prescribed dose	30.6 Gy (range, 21–39.6)
Median lung dose	8.0 Gy (range, 3.5–11.8)
Median lung V20	21% (range, 10–28)

**Abbreviations:** pts, patients; ABVD, adriamycin, bleomycin, vinblastine, dacarbazine; ABVE-PC, adriamycin, bleomycine, vincrisitine, etopside, prednisone, cyclophosophamide; V20, volume of lung receiving at least 20 Gy.

**Figure 1:**
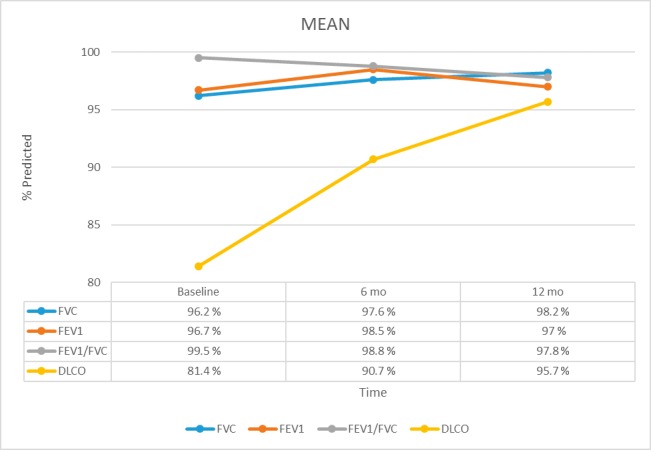
Mean forced vital capacity (FVC), forced expiratory volume in 1 second (FEV_1_), FEV_1_/FVC, and diffusing lung capacity for carbon monoxide (DLCO) predicted percentages at baseline and 6 and 12 months after proton therapy.

The mean forced vital capacity (FVC) was 96.2% ± 16.5% (mean ± SD) predicted at baseline and 98.2% ± 19.4% predicted at 12 months. The mean forced expiratory volume in 1 second (FEV_1_) was 96.7% ± 17.2% predicted at baseline and 97% ± 15.1% predicted at 12 months. The mean FEV_1_/FVC ratio was 99.5 ± 8.29 at baseline and 97.8 ± 8.02 at 12 months. The mean diffusing capacity of the lung for carbon monoxide (DLCO) was 81.4% ± 18.4% predicted at baseline and 95.7% ± 23.5% predicted at 12 months (**Figure 1**).

## Discussion

In this small prospective clinical trial, treatment of mediastinal HL with involved site proton therapy did not result in significant changes in pulmonary function 12 months after treatment. The improved dosimetry correlates with essentially unchanged PFT findings before and after therapy. These results are quite favorable compared with other historical photon studies. In one study of 59 patients who received chemotherapy with or without involved-field photon RT for HL, those patients who received RT had persistently decreased %DLCO at 12 months following completion of RT [8]. In the present study, a likely explanation for the increase in %DLCO from baseline is recovery from chemotherapy-associated toxicity, such as bleomycin [14]. Another study of 38 HL patients who received PFT following completion of involved-field photon RT reported a 13.2% incidence of abnormal FVC and FEV_1_/FVC ratio defined as less than 80% of predicted values [9]. Finally, a prospective study of 30 patients who received mantle-field radiation showed that it took approximately 4 years for the %DLCO to return to baseline [10]. While these studies demonstrated worse pulmonary outcomes, compared with the present study, the likely cause is due to the larger fields that were treated in those studies rather than the use of photons versus protons. Since we do not have a comparative group from our own institution receiving involved-site radiation therapy (ISRT) field radiation with photons, the most relevant comparative data comes from a recent Memorial Sloan Kettering Cancer Center study. The study evaluated PFTs among patients treated with brentuximab + AVD (adriamycin, vinblastine, dacarbazine) ×4 cycles followed by ISRT (photons), and showed no decline in %DLCO of FVC between the pre-ISRT PFTs, post-ISRT PFTs, or the 12-month follow-up [11]. This supports the fact that radiation field size difference probably is of more importance than actual radiation modality. Although the results are part of a prospective study, there are several limitations. The patient population was small and patients were treated heterogeneously with different chemotherapy regimens. Unfortunately, the Hodgkin lymphoma patients came from a number of outside hospitals, which prevented us from restricting enrollment to a homogeneous cohort. Of greatest concern with this factor is the variation in bleomycin doses across patients as it is known to cause pulmonary toxicity. Additionally, we only looked at two follow-up time points. It may be that radiation fibrosis more than 1 year from treatment causes additional pulmonary problems, but our study did not include PFTs as part of long-term follow-up.

While the results of the present study are not surprising, they do confirm that no unexpected changes are observed to the lungs as illustrated through follow-up PFTs. Proton therapy is an important technology that is being used in the management of mediastinal HL. Validation in a larger cohort and longer follow-up are needed.
